# Histopathologic Analysis of the Morpho‐Functional Zones of the Human Acetabular Labrum

**DOI:** 10.1002/ca.70031

**Published:** 2025-09-12

**Authors:** Abdulaziz A. Alomiery, Andrew C. Hall, Thomas H. Gillingwater, Afaf Alsolami, Abduelmenem Alashkham

**Affiliations:** ^1^ Edinburgh Medical School, Department of Anatomy University of Edinburgh Edinburgh UK; ^2^ Basic Science Department, Prince Sultan College for Emergency Medical Services King Saud University Riyadh Saudi Arabia; ^3^ Centre for Discovery Brain Sciences, Biomedical Sciences, the University of Edinburgh Edinburgh UK; ^4^ Central Medical Laboratory and Blood Bank Prince Sultan Military Medical City Riyadh Saudi Arabia; ^5^ Zawia Faculty of Medicine University of Zawia Libya

**Keywords:** acetabular, hip, histopathologic, joint, labrum

## Abstract

The structural and functional adaptation of soft tissues to mechanical load controls their ability to withstand injury and influences their capacity for healing. Similar to the knee meniscus, the acetabular labrum exhibits zonal differences in mechanical load distribution, resulting in distinct regions with unique structural and functional properties. However, little is known about the effect of these zonal adaptations on the severity and distribution of labral degenerative changes. This study aims to assess the impact of labral zonal adaptations on the severity and distribution of histopathologic features. Human tissue was obtained from 9 embalmed cadavers, comprising a total of 16 hemipelves (10 males and 6 females) with an average age of 80 years (age range 66–99). Each hip was divided into 8 distinct regions, resulting in 128 regional segments. Slides were stained using Hematoxylin and Eosin (H&E) and Safranin‐O (Saf O), with the incorporation of fluorescent scanning of eosin (F‐Eosin). Labral histopathologic features were assessed using established modified grading criteria for the knee meniscus. These features were evaluated both globally across the anatomical quadrants of the hip joint and zonally across the inner and outer zones. The global analysis of the labrum revealed a similar distribution of histopathologic features across the superior, anterior, inferior, and posterior quadrants of the hip joint. Conversely, across 128 labral segments, pairwise zonal assessments revealed a significant increase (*p* < 0.05) in the severity of degenerative features, which were predominantly concentrated in the inner labral zone near the articular surface. These degenerative changes encompassed alterations in matrix proteoglycan content, cellularity, collagen organization, and labral articular surface, including the lamellar layer. The increased compactness of labral fibers in the inner zone, minimal vascular penetration, and significant degenerative changes imply that it is a vulnerable area for injury with a potentially limited capacity for healing. The delineation of these distinct zonal frameworks highlights the labrum's functional adaptation to its mechanical environment. The zonal analysis of the labrum provided a considerably more detailed perspective on the distribution dynamics of histopathologic features compared to previous global analyses, offering a more precise understanding of the anatomical factors that may explain zone‐specific vulnerability to injury and degeneration.

## 1 Introduction

The acetabular labrum is a fibrocartilaginous tissue attached to the acetabular rim of the hip joint and has fundamental roles in hip joint stability, with multiple associations to various hip joint pathologies (Byers et al. 1970; Harris et al. 1979; Ueo and Hamabuchi 1984; Safran 2010; Bsat et al. 2016). The labrum experiences tensile and compressive stresses and strains in the circumferential and axial directions due to various ranges of motion at the hip joint (Bharam 2006; Safran 2010). Excessive hip joint movements and increased mechanical stresses have the potential to initiate or exacerbate degenerative changes in the labrum and osteoarthritis (OA) in the joint (Altenberg 1977; McCarthy et al. 2001; Safran 2010). While multiple factors such as aging, labral tear, inflammation, and underlying hip conditions such as femoroacetabular impingement (FAI) can contribute to the onset of labral degenerative changes, they all converge to elevate mechanical stress on the labrum (Lecouvet et al. 1996; Cotten et al. 1998; Abe et al. 2000; Ito et al. 2004; Lewis and Sahrmann 2006; Hunt et al. 2007; Domzalski et al. 2017).

Extensive work by others has demonstrated that the proper formation and maintenance of soft tissues depend on the application of physiologically sufficient mechanical loads (Carter 1987; Di Giancamillo et al. 2017; Polito et al. 2020). In load‐bearing intra‐articular structures such as the knee meniscus and acetabular labrum, different tissue zones experience varying levels of mechanical load (Petersen et al. 2003; Polito et al. 2020). Therefore, distinct zones within the same tissue adopt different structural and compositional characteristics. These inherent variations emerge as functional adaptations to the magnitude of mechanical loads experienced by each zone (Polito et al. 2020). The role of mechanical load has been demonstrated in an in vivo study on immobilized chick embryos (Mikic et al. 2000). The authors demonstrated that the development of synovial joints was impaired when physiological loads were absent (Mikic et al. 2000). For instance, in the absence of mechanical loads, the menisci of the knee joint and the plantar tarsal sesamoid of the tibiotarsal joint did not develop as expected (Mikic et al. 2000). In an animal model of quadriceps contracture syndrome in Doberman puppies, Polito et al. (2020) demonstrated that mechanical load induces early maturation and remodeling of the meniscal extracellular matrix (ECM). However, this process compromises proper collagen fiber organization, leading to disorganized fibers, altered glycosaminoglycan distribution, and reduced biomechanical properties, which collectively weaken the meniscus' ability to withstand compressive forces.

In a variety of soft tissues, such as the knee meniscus, zonal adaptation to mechanical loads has emerged as a key factor in regulating tissue structural development, assessing healing potential, developing tissue engineering strategies, and guiding surgical repair approaches (Nakano et al. 1997; Barber‐Westin and Noyes 2014; Beaufils and Pujol 2017; Yan et al. 2021; Rohde et al. 2023). Moreover, in the context of degeneration, Fuller et al. (2012) demonstrated that meniscal zones influence the mechanisms of meniscal degeneration. They found that the inner zone, being more cartilaginous and richer in glycosaminoglycans (GAGs), is particularly susceptible to proteoglycan degradation by enzymes from the ADAMTS (A Disintegrin And Metalloproteinase with Thrombospondin Motifs) family. In contrast, the outer zone, which is more fibrous with higher collagen content, is more prone to collagen degradation, driven by increased matrix metalloproteinases (MMPs) activity in response to cytokines such as Tumor Necrosis Factor‐alpha (TNF‐alpha). These findings highlight the distinct vulnerabilities of each meniscal zone based on their biochemical composition and exposure to mechanical forces.

Microscopically, different labral zones exhibit structural and compositional differences, including variations in vascular density, collagen type, and proteoglycan content. These changes reflect the labrum's functional adaptation to its mechanical environment (Seldes et al. 2001; Petersen et al. 2003; McCarthy et al. 2003; Kelly et al. 2005). Previous histopathologic attempts to evaluate acetabular labrum degeneration have focused on providing a global analysis relevant to the overall condition of the labral tissue (Domzalski et al. 2017; Kawamura et al. 2021; Battistelli et al. 2023). However, assessing the influence of zonal adaptation on the severity and distribution of labral degenerative properties has been limited in the literature. This may be due to methodological challenges in accurately delineating the boundaries between the inner and outer zones of the labrum, as well as the absence of a histopathologic grading system that allows for zone‐specific assessment.

Therefore, this study aims to (1) develop a reproducible method for dividing the labrum inner and outer zones; (2) systematically assess the severity and distribution of labral histopathological changes across its morpho‐functional zones, defined as regions where tissue morphology has adapted in response to biomechanical forces to fulfill specific mechanical roles; (3) and identify potential zonal vulnerabilities and areas that are affected in the initial stages of degeneration.

## 2 Materials and Methods

### 2.1 Human Hip Joints

**FIGURE 1:**
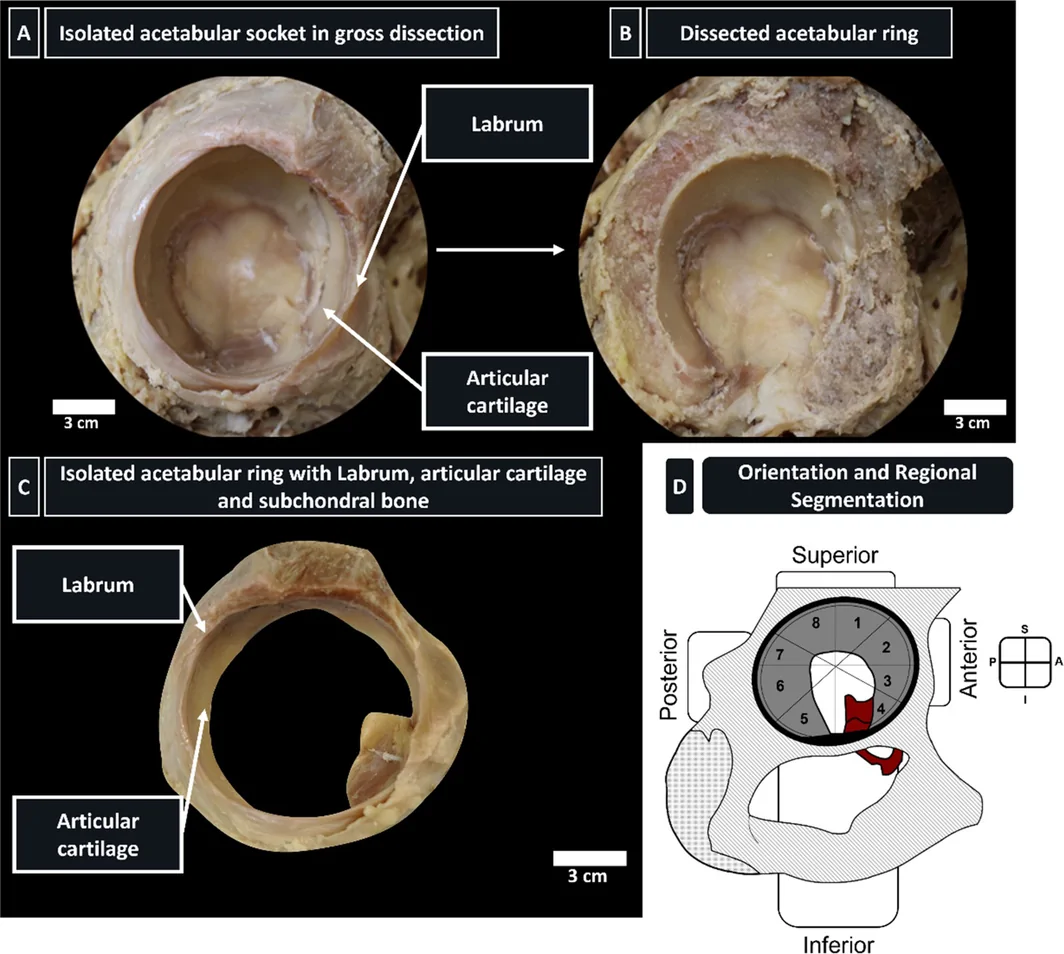
Gross Dissection Procedure. Right male hemipelvis showing the acetabular labrum isolated in a ring shape and segmented into eight regions (1–8). The acetabulum was circumferentially cut through the socket, preserving the labrum, capsular attachment, articular cartilage, and subchondral bone as a single unit (A). Panel (B) shows the residual acetabular bone post‐dissection, while Panel (C) displays the intact acetabular ring. Panel (D) illustrates the anatomical orientation of regions 1–8 and their corresponding quadrants (superior, anterior, inferior, posterior). Cadaveric images used with permission (Ethical approval: ANATED_0033). The scale bar in (A–C) is 3 cm.

A total of 16 hemipelves (10 males, 6 females; mean age: 79.3 years; age range: 63–99 years) were obtained from 9 embalmed cadavers provided by The Department of Anatomy, University of Edinburgh, which is regulated by the Human Tissue (Scotland) Act 2006 guidelines. Ethical approval code: ANATED_0033. Each hemipelvis was dissected to establish a clear visualization of the acetabular labrum, joint capsule, and articular cartilage. For uniform dissection and reproducibility, each hemipelvis was divided into eight acetabular segments using a vertical cut from the posterior end of the transverse acetabular ligament, a horizontal cut at its midpoint, and two diagonal cuts through each quadrant. To ensure consistency between specimens, regional segments were grouped in correspondence to the anatomical regions they represent, where always, segments 8 and 1 represent the superior region, segments 2 and 3 the anterior region, segments 4 and 5 the inferior region, and segments 6 and 7 the posterior region (Figure 1).

### 2.2 Acetabular Labrum Processing and Sectioning

Each of the 8 regional segments obtained was fixed using 10% natural buffered formaldehyde solution (pH =7.4) (Sigma‐Aldrich, Merck Life Science, Gillingham, UK) at 4°C for 24 h. Decalcification was performed using Decalcifying Solution‐Lite (Sigma‐Aldrich). Tissue segments were processed into paraffin wax blocks and stored at 4°C. Each wax block contained a tissue segment with an intact labrum, capsule, cartilage, and bone. Sectioning was performed at 10 μm thickness using Leica RM 2245 microtome (Milton Keynes, UK) and high‐performance microtome blades (MX35 Premier) (Fisher Scientific).

### 2.3 Tissue Staining

Tissues underwent hematoxylin and eosin (H&E) staining to assess cellularity and cellular morphology. Fluorescence scanning of eosin (F‐Eosin) was employed to evaluate collagen alignment and fiber organization and to obtain a general overview of tissue structural morphology (Heo and Song 2011). Safranin O‐Fast Green (Saf‐O) staining facilitated the semi‐quantitative evaluation of proteoglycan content (Pauli et al. 2012). Fluorescence scanning of eosin (F‐Eosin) and normal brightfield microscopy (H&E) were utilized to qualitatively assess collagen degeneration.

### 2.4 Image Analysis

Slides were scanned at 40× magnification and digitally converted into virtual images using the Hamamatsu NDP slide scanner (Hamamatsu Nanozoomer 2.0HT). The slides were then exported at high resolution using the NDP.Viewer software.

### 2.5 Inner and Outer Labral Division

**FIGURE 2:**
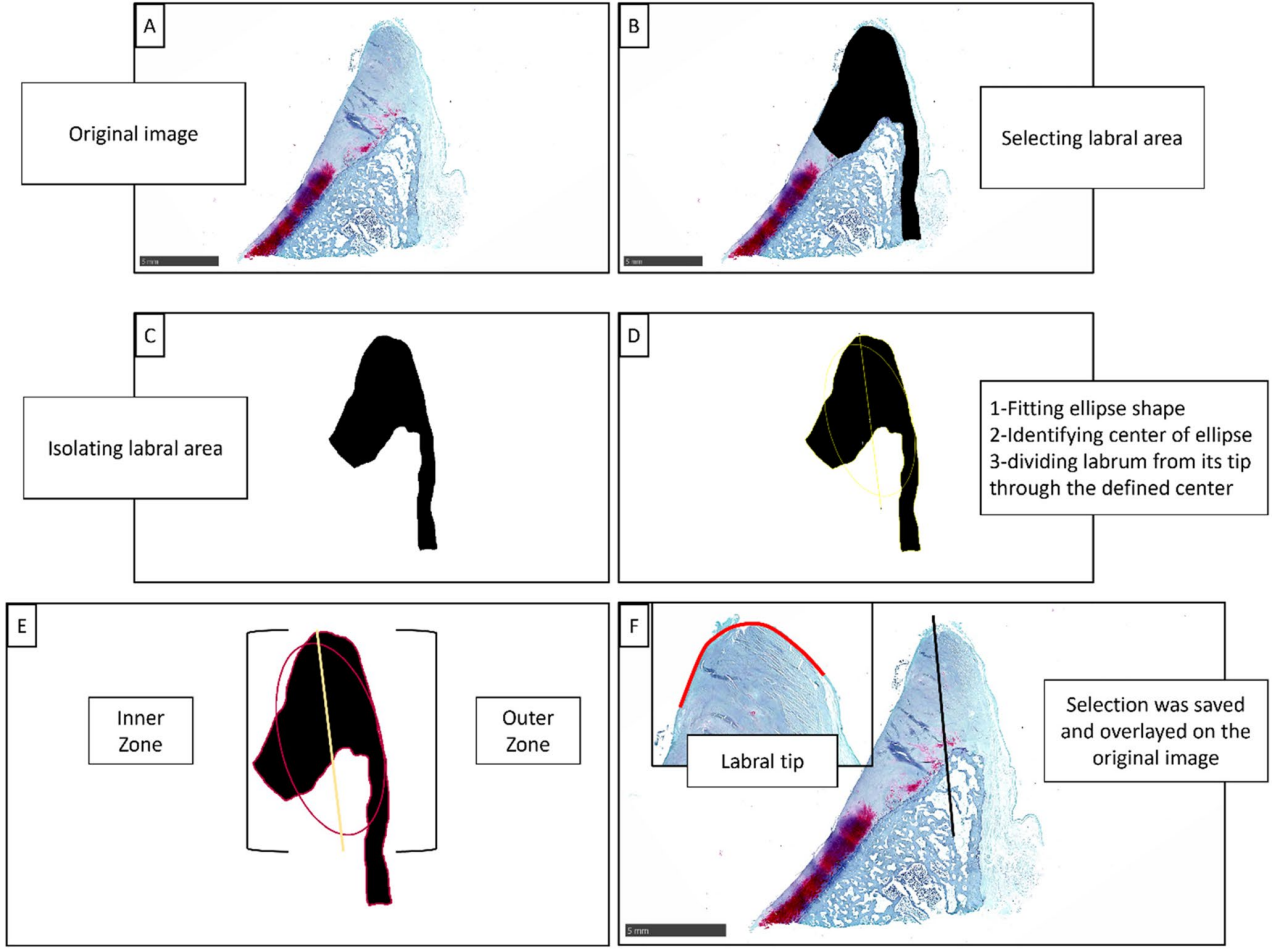
Method of Inner and Outer Labral Division. The flowchart illustrates the analysis steps conducted in the inner and outer labral divisions. Saf O‐stained labral tissue sections were scanned and digitally converted into virtual slides. The workflow developed here was performed using the image analysis software FIJI ImageJ. First, the original image was imported into ImageJ software (A). Second, the labral area (i.e., region of interest—ROI) was manually defined and selected, the selection saved, and the region of interest was colored in black (B). Third, background tissue components were cleared to isolate the region of interest (C). An elliptical shape was fitted over the region of interest to normalize the definition of the labral centroid. The centroid (i.e., centre point of a selection) of the ellipse was regarded as the centre of the selected labral shape. A vertical line using the straight‐line tool was manually drawn from the tip of the labrum (i.e., labral highest point) through its defined centre (D). Panel (E) illustrates the inner and outer labral zones after completing the method. The vertical line selection was saved to be applied over the original image for zonal analysis (F). Scale bars are 5 mm long (A–F).

**FIGURE 3:**
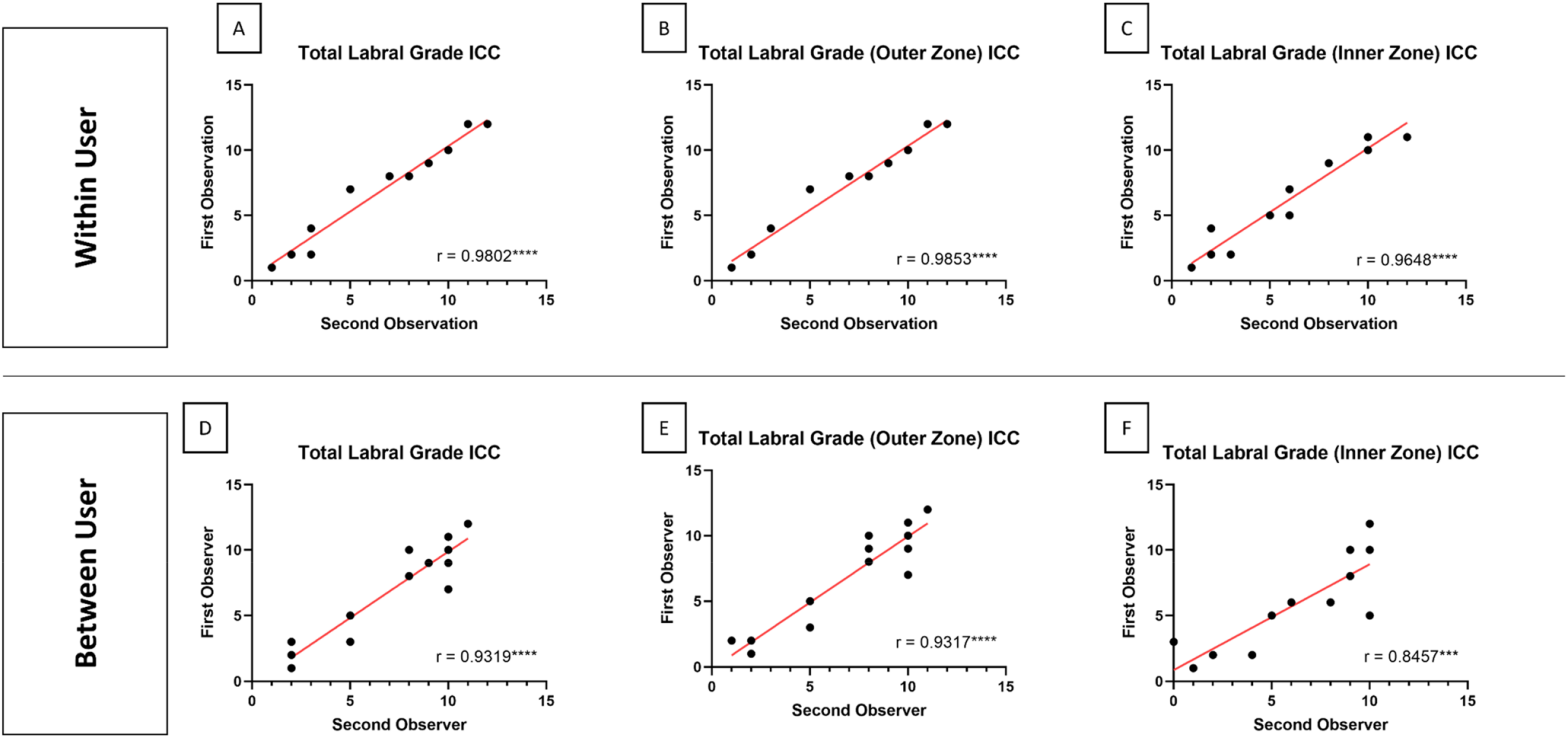
An Overview of the Reliability Tests Conducted (Within Observer and Between Observer) for the Modified Pauli's grading systems of Acetabular labrum Degeneration. Reliability tests were performed on 13 randomly selected samples (10% of the total, *n* = 128), covering the full grading range (0–12). Intra‐rater reliability (within observer) for total labral grade, outer zone, and inner zone was rated “excellent,” with ICC values of 0.980, 0.985, and 0.964, and Cronbach's alpha values of 0.990, 0.993, and 0.982, respectively (A–C). Inter‐rater reliability (between observers) was also “excellent,” with ICC values of 0.931 (total), 0.931 (outer zone), and 0.845 (inner zone), and Cronbach's alpha values of 0.963, 0.963, and 0.916, respectively (D–F).

The workflow developed here was performed using image analysis software *FIJI ImageJ software* (National Institutes of Health, Bethesda, USA). Since labral sections vary in size and shape across the circumference of the hip joint's acetabulum, standardized comparison may be constrained. The developed workflow aimed to reduce variability and provide a more standardized approach for comparison. First, using the *Freehand Selection Tool*, labral borders were manually defined and selected by the primary investigator, then saved as a region of interest (ROI) using the *ROI Manager*. To ensure consistency, the selection process was repeated after multiple time intervals (Figure 2A). Second, the selection was masked in black, and the background was cleared from the remaining tissue components to define the ROI visually (Figure 2B,C). Third, an ellipse shape was fitted on the original labral selection with an overlaying best‐fit ellipse outline, *Edit* > *Selection* > *Fit Ellipse*. The ellipse outline was then saved at the *ROI Manager* (Figure 2D). The centroid of a best‐fit ellipse was used as the reference point for each labral region, offering a stable and reproducible geometric center. Due to the labrum's irregular and variable morphology, standard methods, like bounding boxes in ImageJ, often yielded inconsistent results. In contrast, ellipse fitting—based on the second central moments of the pixel distribution—captures the overall shape and orientation, approximating geometric normality. This approach minimizes variability in centroid placement and enables consistent morphometric comparisons across specimens (Abràmoff et al. 2004; Mulchrone and Choudhury 2004). A vertical line from the labral tip, the superior point of deflection marking the first curvature change, through the previously determined centroid, was drawn using ImageJ's *straight‐line tool* to guide labral zonal division (Figure 2E,F). The labral tip was consistently identifiable across specimens, supported by high intra‐ and inter‐observer reliability in applying the Modified Pauli's grading system (Figure 3). This reference line was saved as an overlay and applied to the original images for consistent zonal analysis across specimens.

### 2.6 Modified Pauli's Grading System

**FIGURE 4:**
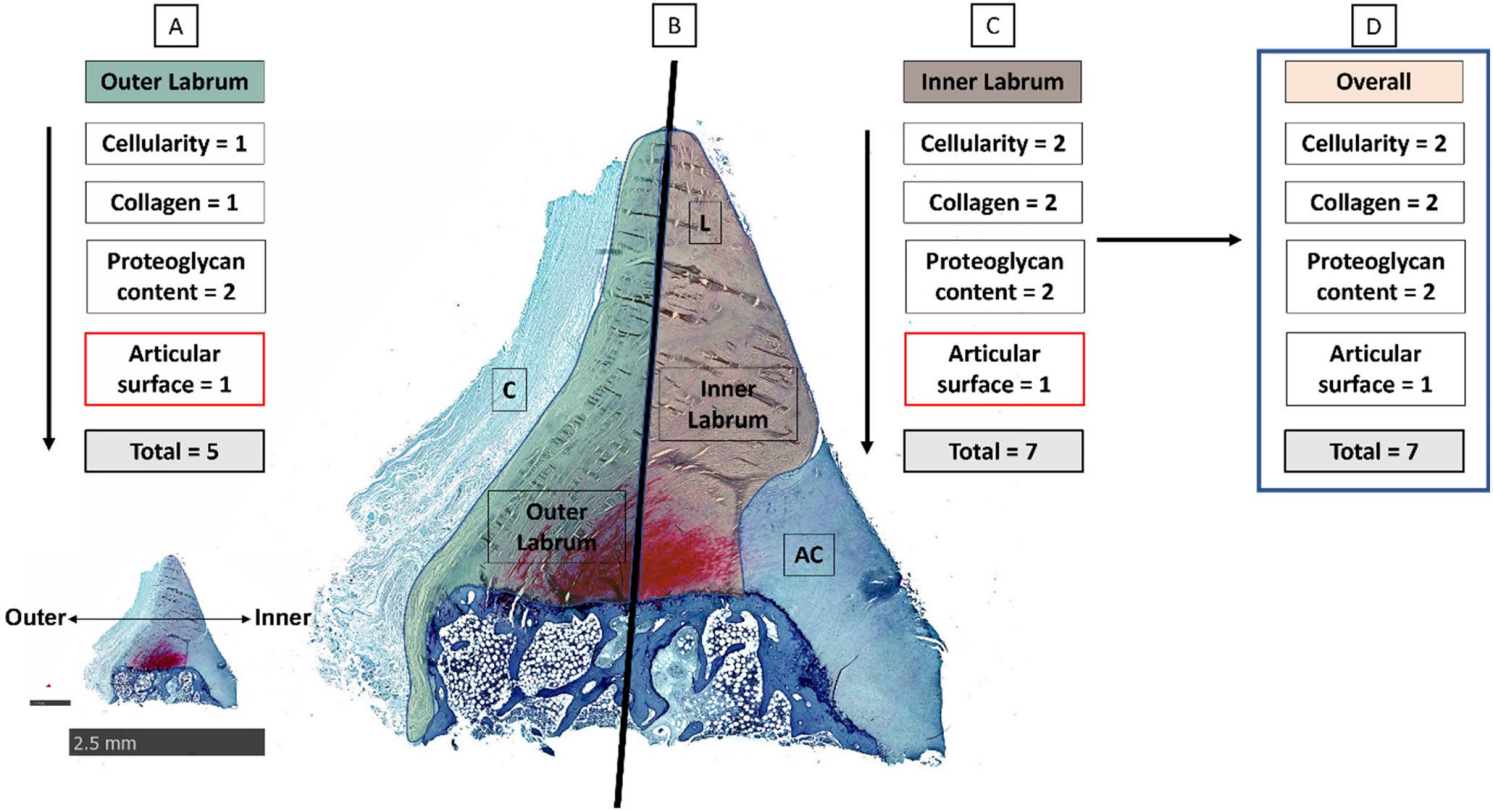
Modified Pauli's Grading System for The Acetabular Labrum. Histological illustration of zonal evaluation for labral degeneration. The outer zone is assessed for cellularity, collagen, and proteoglycan content (A), while the inner zone includes these components in addition to articular surface evaluation (B). As the labrum has a single articular surface (inner zone), the surface score was added as a constant to both zones (Red boxes) to equalize total scores and enable comparison. The overall labral grade reflects the zone with greater severity, which in this example is the inner zone (D). L: Labrum; AC: Articular cartilage; C: Capsule. The scale bar is 2.5 mm.

To systematically evaluate the histopathologic changes of the labrum, Pauli's histological grading system of the knee meniscus (Pauli et al. 2011) was adopted and modified to assess the acetabular labrum at the cross‐sectional level, including its associated zones. The modified Pauli's grading system was established to assess the grade of labral degenerative changes within the following aspects: articular surface, cellularity, collagen organization, and Safranin‐O‐Fast Green matrix staining intensity. The evaluation criteria selected the most severe change within each assessed slide. For example, if different levels of the same degenerative component are present in the same section (e.g., cellularity level 1 and 2), the most severe change was selected (i.e., cellularity level 2). Each zone was evaluated separately, providing a zone‐specific assessment. This approach allowed for assessing the degeneration pattern by evaluating how labral morpho‐functional zones can influence the severity and distribution of degenerative changes. Additionally, the overall labral grade, encompassing both the inner and outer zones, was determined by selecting the score from the zone with the most severe change for each scoring component in the evaluation criteria. For instance, if the score for cellularity was 2 in the inner zone and 3 in the outer zone, then the overall score is 3 (i.e., a greater score in severity). Since the labrum has only one particular surface, which is associated with the inner zone only, the score of the surface analysis component in the Modified Pauli's Grading System was added as a constant value to both zones (Figure 4). This modification ensured that both zones had equivalent total possible scores, allowing for direct comparison between them.

**FIGURE 5:**
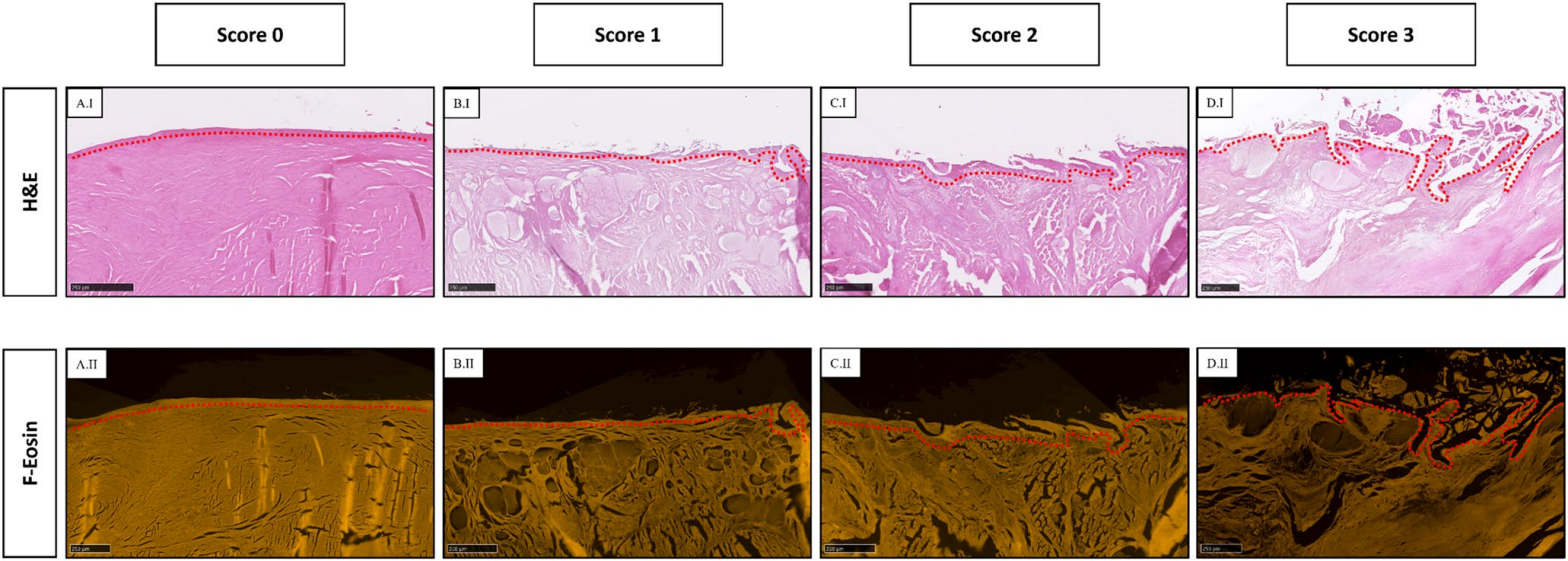
Modified Pauli's Grading System for The Acetabular Labrum: Scoring Criteria of The Labral Articular Surface. Histological illustration related to the articular surface degenerative scores, tissues stained with H&E demonstrated by two scanning representations using brightfield normal‐H&E and fluorescence scanning of eosin F‐Eosin. Brightfield enabled the assessment of tissue features with normal H&E representation, while fluorescence provided sufficient color contrast for detailed tissue feature evaluation. Score 0 (AI‐II) illustrates normal tissue representation with an intact, smooth, non‐frayed articular surface. Score 1 (BI‐II) represents the first stage of tissue changes, characterized by slight fibrillation or slightly undulating of the articular surface. Score 2 (CI‐II) shows moderate fibrillation or markedly undulating articular surface. Score 3 (DI‐II) shows severe fibrillation or disruption of the articular surface with the marked appearance of surface fissures, severe fraying and/or tears. All scale bars = 250 μm.

**FIGURE 6:**
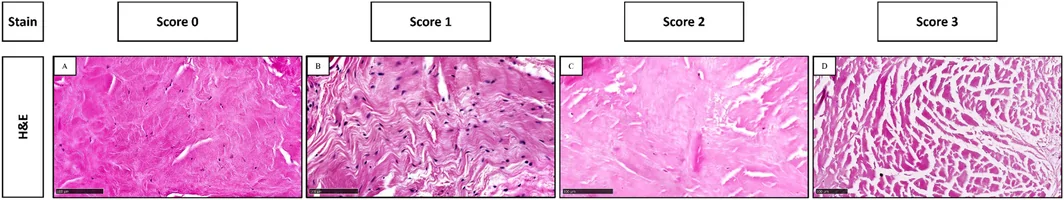
Modified Pauli's Grading System for Acetabular labrum: Scoring Criteria of the Labral Cellularity. Histological illustrations of degenerative changes of labral cellularity, tissues stained with H&E, demonstrating scores associated with increased levels of severity. Score 0 (A) illustrates normal tissue representation with typical cellular amount and regular distribution. Score 1 (B) is characterized by diffuse hypercellularity. Score 2 (C) labral tissue demonstrates diffuse hypo‐cellular to acellular regions. Score 3 (D) is characterized by hypocellularity of labral tissue associated with empty lacuna, marked presentation of cellular death. All scale bars = 100 μm.

**FIGURE 7:**
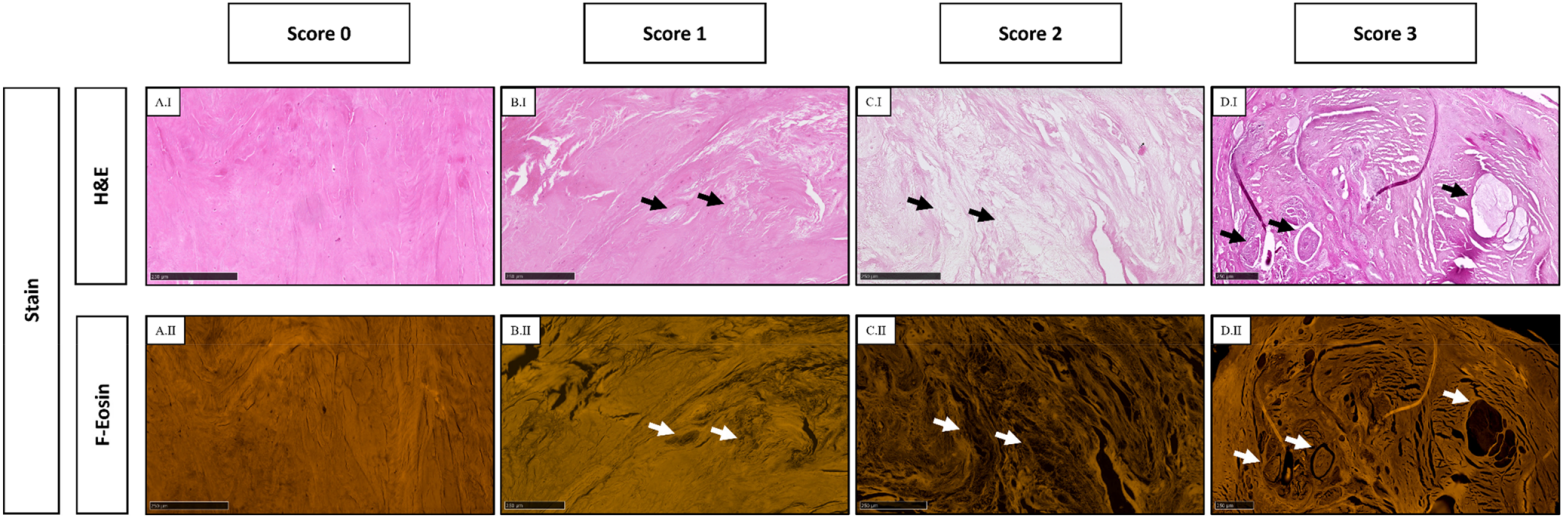
Modified Pauli's Grading System for Acetabular Labrum, Scoring Criteria of Labral Collagen Organization. Histological illustrations of degenerative changes related to labral collagen organization/alignment and fiber organization, tissues stained with H&E demonstrated by two scanning representations using brightfield normal‐H&E and fluorescence scanning of eosin F‐Eosin. Brightfield enabled the assessment of tissue features with normal H&E representation, while fluorescence provided sufficient color contrast for detailed tissue feature evaluation. Score 0 (AI‐II) illustrates normal tissue representation with organized collagen fibers and homogenous eosinophilic staining of the extracellular matrix. Score 1 (BI‐II) is characterized by mostly organized collagen fibers, and diffuse foci of hyaline or mucinous degeneration (Black arrows‐H&E) (White arrows‐F‐Eosin). Score 2 (CI‐II) is characterized by unorganized collagen fibers, confluent foci or bands of hyaline or mucinous degeneration and fraying of fibrocartilaginous labral tissue (Black arrows‐H&E) (White arrows‐F‐Eosin). Score 3 (DI‐II) is characterized by unorganized collagen fibers, fibrocartilaginous separation (oedema, cyst formation), severe fraying and tears (Black arrows‐H&E) (White arrows‐F‐Eosin). All scale bars = 250 μm.

**FIGURE 8:**
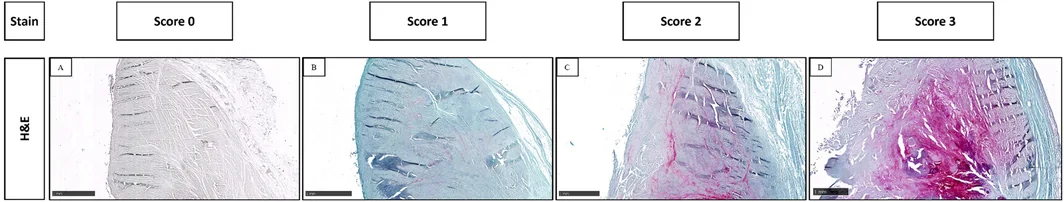
Modified Pauli's Grading System for Acetabular Labrum, Scoring Criteria of Labral Matrix Staining (Proteoglycan Content). Histological illustrations of degenerative changes related to proteoglycan content associated with matrix staining intensity of Saf O. Score 0 (A) normal tissue representation with no staining for Saf O. Score 1 (B) represents a slight increase in proteoglycan content indicated by slight staining intensity for Saf O. Score 2 (C) illustrates a moderate increase in proteoglycan content indicated by moderate staining intensity for Saf O. Score 3 (D) illustrates a marked increase in proteoglycan content indicated by strong staining intensity for Saf O. All scale bars = 1 mm.

One of the important modifications applied to the adopted Pauli's Grading System was the incorporation of F‐Eosin in addition to routine H&E staining for the articular surface and collagen evaluation (Figures 5 and 7). Tissues stained with H&E were evaluated in both brightfield normal‐H&E and fluorescence scanning of eosin F‐Eosin. In the brightfield scanning mode, tissue features were assessed following normal H&E staining, while fluorescence scanning was utilized to enhance color contrast and enable detailed evaluation of tissue features. See (Figures 5, 6, 7, 8) for examples of the evaluation components and their related severity ranges in both modes. The range of possible total scores for the acetabular labrum was 0 to 12, which was further categorized into four grades: G1 = 0 to 2, G2 = 3 to 5, G3 = 6 to 8, and G4 = 9 to 12. Grade 1 represents normal tissue changes, Grade 2 indicates mild degeneration, Grade 3 indicates moderate degeneration, and Grade 4 indicates severe degeneration (Table 1).

**TABLE 1:** Histological assessment and criteria of labral degenerative changes.

Surface including lamellar layer (Figure 5)	Score
Smooth	0
B Slight fibrillation or slightly undulating	1
C Moderate fibrillation or markedly undulating	2
D Severe fibrillation or disruption	3
Cellularity (Figure 6)	Score
Normal^a^	0
B Diffuse hypercellularity	1
C Diffuse hypo/acellular regions	2
D Hypocellularity (empty lacuna, pycnotic cells)	3
Collagen organization/alignment and fiber organization (Figure 7)	Score
Collagen fibers organized, homogenous eosinophilic staining of extracellular matrix	0
B Collagen fibers organized, diffuse foci of hyaline or mucinous degeneration	1
C Collagen fibers, unorganized, confluent foci or bands of hyaline or mucinous degeneration, fraying	2
D Collagen fibers unorganized, fibrocartilaginous separation (oedema, cyst formation), severe fraying and tears	3
Matrix staining (Safranin‐O‐Fast Green) (Figure 8)	Score
None	0
B Slight	1
C Moderate	2
D Strong	3

*Note:* This table illustrates the range of possible total scores for each labral regional segment (0–12), which was further categorized into 4 grades: G1 = 0–2, G2 = 3–5, G3 = 6–8, and G4 = 9–12. Grade 1 represents normal tissue changes; Grade 2 is mild degeneration; Grade 3 is moderate degeneration and Grade 4 is severe degeneration.

^a^ Normal cellularity refers to an appropriate balance and distribution of cells without evidence of abnormal proliferation, depletion, or significant cell clustering, indicating tissue homeostasis and proper functioning. See Schon et al. (2020).

### 2.7 Validation of the Grading Systems

To ensure minimal bias and enhance the reliability of the findings, inter‐ and intra‐rater agreement was performed using the intraclass correlation coefficient (ICC) for single measures of absolute agreement and Cronbach's alpha. This analysis was conducted using the SPSS version 24 statistical package (IBM, Armonk, USA). The inter‐ and intra‐rater reliability for the Modified Pauli's grading systems of the acetabular labrum was “Excellent” for the total labral grade and total grade of the outer and the inner zones (*r* values > 0.90) (Koo and Li 2016) (Figure 3). Whereas the inter‐rater reliability for the total labral grade for the inner zone was “Good” (*r* value = 0.845) (Figure 3).

### 2.8 Statistical Analysis

Data were collected and tabulated in Excel 2022 (Microsoft, Redmond, USA). GraphPad Prism version 9.4.1 (681) (GraphPad Software Inc., San Diego, USA) was used for all graphical representations. SPSS version 24 (IBM, Armonk, USA) statistical package was used for all statistical data analysis. Wilcoxon signed‐rank test for paired data was conducted for the pairwise analysis of degenerative changes between the inner and outer zones of the labrum. This was to evaluate the effect of observed zonal disparities on the outcomes of total labral grade and the underlying scoring components of the Modified Pauli's Grading System. The data representation for the Wilcoxon signed‐rank test is presented as counts of severity ranks, and the level of statistical significance is highlighted as *p* < 0.05.

Degeneration severity grades used for the quadrant‐based Chi‐square analysis were derived by combining the inner and outer zone scores into a single overall value for each labral segment. These values were then grouped by anatomical quadrant to assess distribution patterns of degeneration. The difference in labral grade of degenerative changes across hip joint anatomical quadrants was evaluated using the Chi‐square test of independence and Bonferroni post hoc analysis. The data representation for Chi‐square is reported as counts and proportions, Chi‐square value (χ^2^), degree of freedom (df) and sample size (n). The level of *p < 0.05* was considered statistically significant. Tissue segments of each level of labral degenerative changes were assigned to their corresponding anatomical quadrants (superior, anterior, inferior and posterior).

## 3 Results

To capture a more detailed understanding of labral histopathologic changes, this study compared global and zonal analyses to investigate the distribution of degenerative changes (normal, mild, moderate, severe) across the hip joints, aiming to identify subtle variations that might otherwise be overlooked.

### 3.1 Differences Across Hip Joint Anatomical Quadrants

**FIGURE 9:**
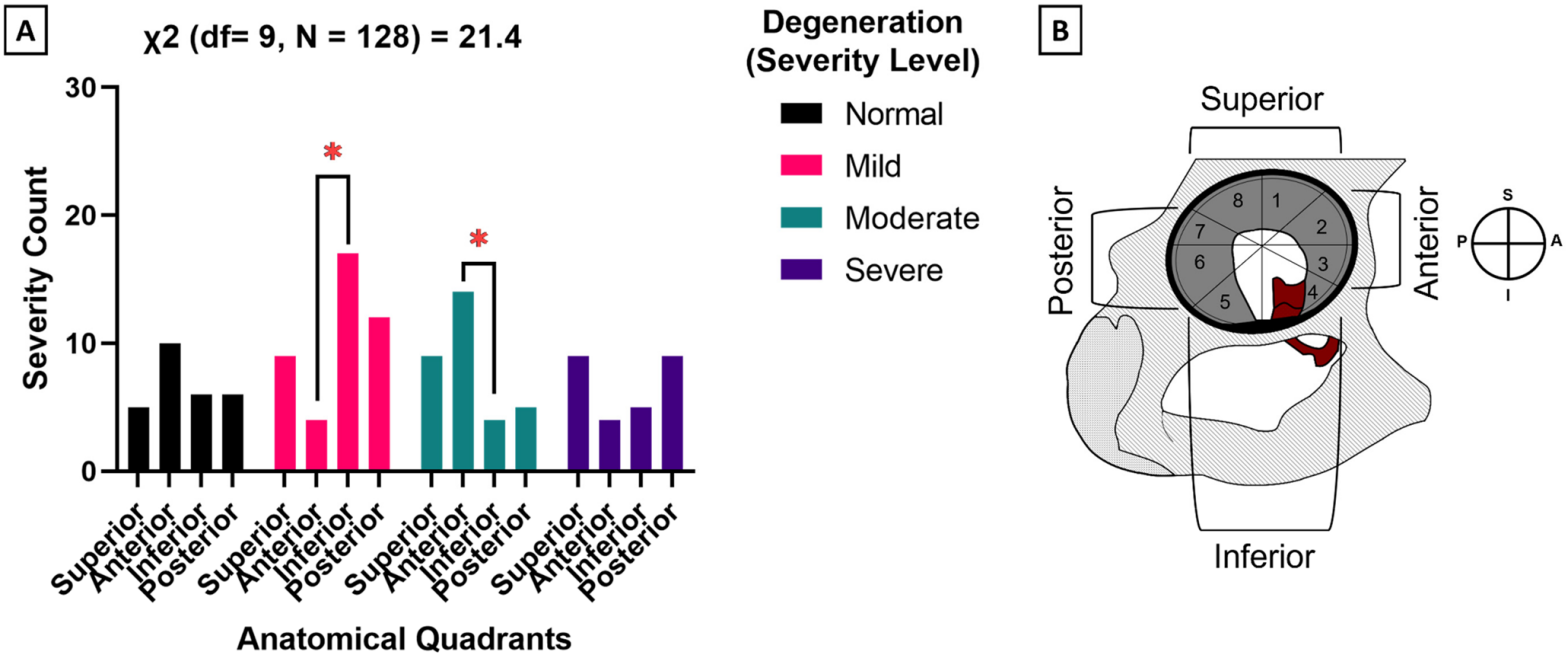
Differences in Labral Degeneration Levels (Modified Pauli's Grading System) Across Hip Joint Anatomical Quadrants. Bar graph illustrating Chi‐square and post hoc *z*‐tests (with Bonferroni correction) assessing differences in Modified Pauli's degeneration grades across hip joint quadrants. Bars represent counts of labral segments per severity level, with colors indicating degeneration grade. Degeneration differed significantly across quadrants, χ^2^ (9, *N* = 128) = 21.4, *p* = 0.011. Mild and moderate degeneration levels showed significant differences between anterior and inferior quadrants, *p* < 0.05 (A). Panel (B) shows anatomical quadrant orientation.

In the normal labral tissue changes group (*n* = 27), the anterior anatomical quadrant showed the highest proportion of tissue segments classified with normal tissue changes (10/27, 37%), suggesting that this quadrant was the least affected by degenerative changes. In contrast, the superior quadrant had the lowest proportion of tissue segments with normal labral tissue (5/27, 18.5%). The inferior and posterior quadrants both exhibited a consistent distribution of normal labral segments, with 6/27 (22.2%) in each quadrant. There were no statistically significant differences in the distribution of tissue segments with normal labral tissue changes (Figure 9, Tables 2 and 3).

**TABLE 2:** Chi‐Square cross‐tabulation of modified Pauli's grading system (labral degeneration) levels across hip joint anatomical quadrants.

			Anatomical quadrants				Total
			Superior	Anterior	Inferior	Posterior	
Labral Degeneration Group	Normal	Count	5_a_	10_a_	6_a_	6_a_	27
		% within Labral Degeneration Group	18.5%	37.0%	22.2%	22.2%	100%
	Mild	Count	9_a,b_	4_b_	17_a_	12_a,b_	42
		% within Labral Degeneration Group	21.4%	9.5%	40.5%	28.6%	100%
	Moderate	Count	9_a,b_	14_b_	4_a_	5_a,b_	32
		% within Labral Degeneration Group	28.1%	43.8%	12.5%	15.6%	100%
	Severe	Count	9_a_	4_a_	5_a_	9_a_	27
		% within Labral Degeneration Group	33.3%	14.8%	18.5%	33.3%	100%
Total		Count	32	32	32	32	128
		% within Labral Degeneration Group	25.0%	25.0%	25.0%	25.0%	100.0%

*Note:* The following table illustrates the counts and proportions of the difference of modified Pauli's Grading System (labral degeneration) levels across Hip Joint Anatomical Quadrants with post hoc analysis involving pairwise comparisons using the z‐test of two proportions with a Bonferroni correction. Each subscript letter denotes a subset of anatomical quadrant categories whose column proportions do not differ significantly from each other at the *p* < 0.05.

**TABLE 3:** Chi‐Square tests of difference of modified Pauli's grading system (labral degeneration) levels across hip joint anatomical quadrants.

	Value	df	Asymptotic significance (2‐sided)
*Pearson Chi‐Square*	21.485	9	0.011
Likelihood Ratio	21.839	9	0.009
Linear‐by‐Linear Association	0.512	1	0.474
No of Valid Cases	128		

*Note:* The table illustrates Chi‐square results, degrees of freedom (df) and significance at *p* < 0.05. Chi‐Square degree of freedom (df) = (*r*−1) (*c*−1).

In the mild labral degenerative changes group (*n* = 42), the inferior anatomical quadrant showed a statistically significant higher proportion of tissue segments (17/42, 40.5%) compared to the anterior quadrant (4/42, 9.5%) (*p* < 0.05). The distribution was consistent across the other quadrants, with the anterior quadrant having the lowest proportion of mild degenerative changes (4/42, 9.5%). The superior and posterior quadrants exhibited similar proportions, with 9/42 (21.4%) and 12/42 (28.6%) of tissue segments, respectively.

In the moderate labral degenerative group (*n* = 32), the proportion of tissue segments classified with moderate degenerative changes was significantly higher in the anterior anatomical quadrant (14/32, 43.8%) compared to the inferior quadrant (4/32, 12.5%) (*p* < 0.05). On the other hand, no significant differences were found within other quadrants. The superior quadrant had the second highest proportion (9/32, 28.1%), while the inferior and posterior quadrants exhibited similar proportions, with 4/32 (12.5%) and 5/32 (15.6%), respectively (Tables 2 and 3, Figure 9).

In the severe labral degenerative group (*n* = 27), the proportion of tissue segments was the same in both the superior and posterior quadrants, with 9/27 (33.3%) in each. The anterior and inferior quadrants displayed similar tissue segment proportions, with 4/27 (14.8%) and 5/27 (18.5%), respectively. *Post hoc* analysis revealed no statistically significant differences in the distribution of tissue segment proportions with severe labral degenerative changes across all quadrants (Figure 9, Tables 2 and 3).

Collectively, the distribution of degeneration across anatomical quadrants revealed some variation between groups. However, no consistent spatial pattern of degeneration was observed across all levels. While certain quadrants showed statistically significant differences within specific degeneration categories, these differences did not follow a unified or directional trend.

### 3.2 Histopathologic Analysis of the Labrum Across Morpho‐Functional Zones

Developing a robust classification system was essential for understanding the pathological progression of labral degeneration and its impact on joint function. To investigate the effect of zonal adaptation on the distribution of labral degeneration, 128 labral segments were evaluated. For each segment, a pairwise zonal assessment was conducted to examine the distribution of the degenerative changes scoring components (i.e., matrix quality, cellularity, and collagen organization), as well as the total labral grade between the inner and outer zones.

**FIGURE 10:**
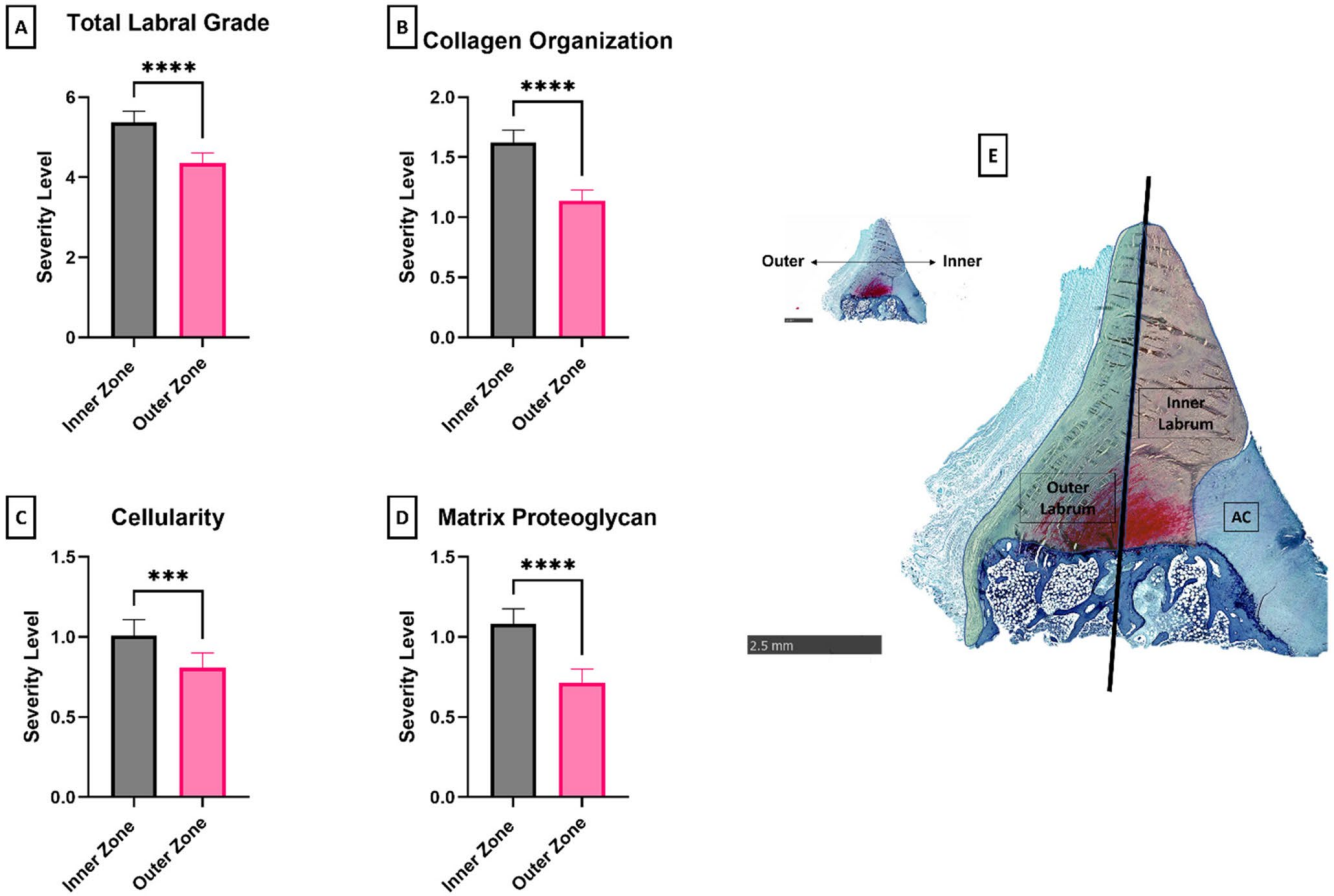
Difference of Modified Pauli's Grading System Scoring Components Between the Inner and Outer Labral Zones. Wilcoxon Signed‐Rank and Exact Sign Tests were used to assess inner and outer labral zone differences in total degeneration grade and individual components (matrix, cellularity, collagen organization). Of 128 segments, total grade severity was higher in the inner zone in 71 segments, compared to 7 in the outer zone *p* < 0.005 (A). Collagen disorganization was greater in 45 inner segments, compared to 9 outer segments *p* < 0.005 (B). Increased cellularity was observed in 24 inner segments, compared to 5 outer segments *p* = 0.001 (C). Matrix loss was found in 41 inner segments, compared to 7 outer segments *p* < 0.005 (D). Panel (E) shows a histological cross‐section indicating inner (light red) and outer (light green) zones stained with Saf O. AC: Articular cartilage.

In the collagen assessment, severe degenerative features—including unorganized collagen fibers, fibrocartilaginous separation (edema, cyst formation), severe fraying, and tears—were significantly more pronounced in the inner labral zone, affecting 45 segments (*p* < 0.05), compared to 9 segments in the outer labral zone (Figure 10). The severe degeneration within the inner zone suggests a higher exposure to mechanical forces, rendering it comparatively more vulnerable to degeneration and repetitive mechanical load. The matrix evaluation showed a significant increase in degeneration severity in the inner zone, as indicated by higher proteoglycan content accumulation. This was observed in the inner labral zone in 41 segments (*p* < 0.05), compared to 7 segments in the outer labral zone (Figure 10). Complemented by the increased degeneration within collagen fibers in the inner zone, the higher proteoglycan content observed suggests an increase in compressive mechanical forces within the inner zone compared with the outer zone. For cellularity, the assessment revealed a significant increase in the severity of degeneration in the inner labral zone, characterized by more regions with diffuse hypocellularity and acellularity, as well as signs of cell death, such as empty lacunae and pyknotic cells. This was evident in 24 segments in the inner labral zone (*p* = 0.001), compared to 5 segments in the outer labral zone. In 95 segments, there was no difference, with both zones showing the same severity of degeneration (Figure 10). The levels of cellular degeneration observed within the inner zone complement the changes in matrix quality and collagen organization, collectively indicating a higher susceptibility to injury and degeneration due to increased exposure to mechanical forces compared to the outer zone.

The total labral grade, which is the sum of all scoring components, showed a significant increase in the severity of degenerative changes in the inner labral zone for 71 segments (*p* < 0.005), compared to only 8 segments in the outer labral zone. Meanwhile, 49 segments showed no difference, having the same severity score in both zones (Figure 10). Collectively, the evaluation of the degenerative components suggests an increased vulnerability to repetitive mechanical load within the inner zone. In contrast, the outer zone benefited from its relatively distant location from the mechanical forces exerted by the femoral head. Therefore, labral zonal adaptation played an important role in mapping the histopathologic features, highlighting how tissue structural organization contributes to potentially inherent tissue vulnerabilities.

## 4 Discussion

While several studies have addressed the functional adaptation of meniscal zones to mechanical loads (Fuller et al. 2012; Barber‐Westin and Noyes 2014; Polito et al. 2020), limited information is available in the literature on the zonal adaptation of the acetabular labrum. The findings presented in this study offer the first standardized zonal analysis of the labrum, providing a novel method for dividing the labrum into its inner and outer zones, where the boundaries of each zone are systematically defined. The incorporation of the developed zonal division method into the semi‐quantitative assessment of tissue degenerative properties (i.e., Modified Pauli's Grading System) allowed for consistent mapping of tissue changes. The current findings highlight the importance of zonal analysis over global analysis in identifying areas of the tissue that might be sensitive to mechanical injury or degeneration. Furthermore, the observation in the current study indicates that the labral morpho‐functional zones exhibit features similar to the functional adaptations observed in other soft tissues such as the meniscus of the knee, glenoid labrum, and the triangular fibrocartilage complex of the wrist (Arnoczky and Warren 1982; Bednar et al. 1991; Cooper et al. 1992).

In the knee meniscus and acetabular labrum, the shape and anatomical positioning, including both their regional location and structural relationships, lead to different exposure to mechanical forces across the main body of the tissue (Petersen et al. 2003; Polito et al. 2020). Therefore, distinct zones within the same tissue develop different structural and compositional characteristics. These variances emerge as functional adaptations to the magnitude of mechanical forces experienced by each zone (i.e., morpho‐functional zones) (Polito et al. 2020). In the knee meniscus, three morpho‐functional zones that differ in structural and compositional properties can be identified: (1) the red‐red zone, rich in vascularity and directly connected to the joint capsule; (2) the white‐white zone, devoid of blood vessels and aligned with the inner sharp edge of the meniscus; and (3) an intermediate red‐white zone, exhibiting intermediate vascularisation and cell phenotyping characteristics (Di Giancamillo et al. 2017). Petersen et al. (2003) provided the first insights into labral zonal adaptation. Similar to the meniscus, the current study demonstrates that the acetabular labrum can be divided into two zones: capsular zone (load‐distant) and articular zone (load‐bearing). The outer, well‐vascularised, dense connective tissue zone is located externally towards the joint capsule (i.e., outer zone) and contains types I and III collagen. The fibrocartilaginous zone, on the other hand, is located internally, towards the articular surface (i.e., inner zone), and contains type II collagen with a marked reduction in vascularity (Petersen et al. 2003). Furthermore, the current study shows that in the labrum, the stimulus for the development of fibrocartilage is provided by intermittent compressive and shearing forces, while dense connective tissue is formed as a functional adaptation to tensional stress. The current observations confirm and expand, with a detailed systematic analysis, on the initial observations provided by Petersen et al. (2003). These observations provided the foundation for understanding how mechanical load shapes the normal structural organization of each zone within load‐bearing fibrocartilaginous tissues. Building on this, the current study continues to explore how these zonal adaptations influence the severity and distribution of labral degenerative changes.

Previous histopathologic studies aimed at evaluating acetabular labrum degeneration have primarily focused on features relevant to the overall condition of the labral tissue (i.e., global analysis). While this approach is important, it has not addressed the potential influence arising from disparities within the labrum's functional adaptation (Domzalski et al. 2017; Kawamura et al. 2021; Battistelli et al. 2023). Therefore, to achieve a thorough understanding of the distribution dynamics of labral histopathologic changes, the zonal division method developed in this study was incorporated into the histopathologic analysis of the labrum. In the current study, the global analysis of the labrum showed a relatively consistent distribution of grades of histopathologic changes, with a few distinct differences observed, but no clear pattern emerging across the quadrants (Figure 9). The proportion of tissue segments classified as normal, mild, moderate, and severe was similarly distributed across the anatomical quadrants. Slight differences in distribution were observed in the proportion of tissue segments classified with mild labral degenerative changes, which were highest in the inferior quadrant (17/42, 40.5%) (Figure 9). Additionally, the proportion of tissue segments classified with moderate labral degenerative changes was the highest in the anterior anatomical quadrant (14/32, 43.8%) (Figure 9). Nevertheless, while the global analysis showed a relatively consistent distribution, the cross‐sectional examination revealed significantly distinct zonal differences. At the cross‐sectional level of the labrum, the analysis addressed the vulnerability of certain zonal territories to degeneration.

The current investigation revealed a statistically significant increase in the severity of histopathologic changes in the inner zone, indicating a potential vulnerability to injury and degeneration. The pattern of degeneration showed a progressive increase from the outer to the inner zone. This trend was evident in the total labral grade (i.e., the sum of all scoring components) and within scoring components (matrix, collagen, and cellularity) (Figure 10). The importance of zonal analysis over global analysis in understanding tissue degeneration cannot be overstated. Similar to the zonal analysis of the knee meniscus by Fuller et al. (2012), in which the acetabular labrum also revealed distinct biochemical and cellular responses to degenerative stimuli that might have been obscured by a global approach.

The significance of labral injury and degeneration arises from its association with the progression of joint osteoarthritis (OA), influenced by multiple degenerative processes such as inflammation and normal ‘wear and tear’ (McCarthy and Busconi 1995; Domzalski et al. 2017). Furthermore, as joints age and experience prolonged mechanical use, labral degeneration and associated labral tears can occur (Lecouvet et al. 1996; Cotten et al. 1998; Abe et al. 2000; Lewis and Sahrmann 2006; Hunt et al. 2007). Outlining labral degeneration and healing, Domzalski et al. (2017) investigated labral histopathologic features in late‐stage OA, regardless of the specific zone. They identified chondrocyte apoptosis as a key factor in matrix degeneration, with cellular apoptosis being observed in 15 specimens and consistently associated with significant tissue degeneration. In areas of apoptosis, the matrix appeared hypochromatic, disorganized and weakened. A similar observation was noted in the current study, with the severity of cellular and collagen fiber degeneration being significantly higher in the inner zone (Figures 6 and 7). This was evident from the observed hypocellularity, which was associated with empty lacunae and pyknotic cells (Figure 6). Additionally, in the current study, disorganized collagen fibers, fibrocartilaginous separation, and associated features such as oedema and cyst formation were significantly more prevalent in the inner zone. These findings highlight the progressive degeneration of the labrum and its potential association with conditions such as OA and hip dysplasia. Moreover, Domzalski et al. (2017) highlighted not only the degenerative processes but also the healing mechanisms involved. They observed that the presence of blood vessels was linked to the long‐standing degeneration of the labral matrix, especially in dysplastic hips where the incongruent hip joint had impinged on the labrum over an extended period of time. Notably, most of the blood vessels were located near the capsule, but they were also found within the substance of the labrum, particularly adjacent to the labral tear (Domzalski et al. 2017). These observations align with previous reports (Petersen et al. 2003), highlighting the distinct distribution of labral properties and their specific zonal localisation.

The zonal framework in the current study offers insight into the underlying mechanisms within specific zones that potentially influence labral healing capacity and its associated vascular distribution. The significant increase in matrix proteoglycan content observed in the inner zone reflects its increased exposure to compressive mechanical loads (Solis‐Cordova et al. 2023). Fibrocartilaginous tissues are commonly acknowledged for their limited blood supply (Benjamin and Evans 1990; Koch and Tillmann 1995; Petersen et al. 2002). Adaptation to compressive and shear forces promotes a more cartilaginous and compact structure to withstand pressure (Benjamin et al. 2006). Consequently, with long‐term exposure to mechanical forces, degeneration and tear progression occur at an increased rate and severity within the minimally vascularised inner zone. This is due to its higher fibrocartilage content and collagen type II, which constrain blood vessel penetration through its compact tissue structure.

These observations support the reported structural characteristics of the inner zone of both the labrum and the meniscus, which demonstrate minimal vascularity and increased proteoglycan content, in contrast to the outer zone (Seldes et al. 2001; Petersen et al. 2003; McCarthy et al. 2003; Kelly et al. 2005). Moreover, these findings are similar to the knee meniscus in the distribution and severity of degenerative changes across different zones. In the knee meniscus, as shown in the findings of Fuller et al. (2012), degeneration is more pronounced in the meniscal inner zone, which is rich in glycosaminoglycans (GAGs) and expresses higher levels of aggrecan (ACAN) and type II collagen (COL2A1). The meniscal inner zone undergoes significant aggrecanolysis due to ADAMTS enzyme activity. Conversely, the outer meniscal zone, which is more fibrous and characterized by higher levels of type I collagen (COL1A1) and versican (VCAN), exhibits increased collagenolysis driven by matrix metalloproteinases (MMPs), particularly in response to cytokines such as Tumor Necrosis Factor‐alpha (TNFα) (Fuller et al. 2012). This pattern mirrors the current observations, where the inner labral zone shows a significant increase in degeneration evident from alterations within the tissue matrix, cellularity, and collagen fibers. The current findings agree with those of the meniscus, where degeneration is more pronounced in the inner zone with higher proteoglycan content and minimal vascularity. Similarly, the inner labral zone shows significant degeneration in the matrix, cellularity, and collagen structure. This highlights comparable degeneration patterns in both the labrum and meniscus, suggesting similar responses to mechanical stress. Nevertheless, to fully understand the role of labral morpho‐functional zones in aging, osteoarthritis, and other inflammatory joint conditions, it is essential to provide a histological description at various stages of the disease and across multiple age groups. The current study focused on analyzing the distribution of labral histopathologic changes in an older cadaveric population, which presents a limitation in establishing broader associations across other joint conditions.

## 5 Conclusion

The purpose of the current study was to investigate the relationship between the distribution of degenerative changes in the labrum and the inherent structural differences within its morpho‐functional zones. The integration of the newly developed zonal division approach into the semi‐quantitative assessment of tissue degenerative properties (i.e., Modified Pauli's Grading System) enabled consistent mapping of tissue changes. The findings provided novel insights into the severity and progression of labral histopathologic changes, highlighting specific zonal vulnerability within the inner zone to degeneration due to the inherent structural adaptations of the labral tissue. The inner zone's vulnerability arises from its functional adaptation to compressive and shear forces, promoting a more cartilaginous and compact structure that limits vascular penetration. This makes it potentially more sensitive to greater degenerative changes during aging and pathology. The inherent structural adaptations within the labrum demonstrate its similarities with other fibrocartilaginous tissues, such as the knee meniscus. Zonal analysis of the labrum offers more detailed insights than global analysis, helping to elucidate the anatomical factors that may explain structural disparities within the tissue.

## Conflicts of Interest

The authors declare no conflicts of interest.

## References

[ca70031-bib-0001] Abe, I. , Y. Harada , K. Oinuma , et al. 2000. “Acetabular Labrum: Abnormal Findings at MR Imaging in Asymptomatic Hips.” Radiology 216, no. 2: 576–581. 10.1148/radiology.216.2.r00au13576 10924588

[ca70031-bib-0002] Abràmoff, M. D. , P. J. Magalhães , and S. J. Ram . 2004. “Image Processing With ImageJ.” Biophotonics International 11, no. 7: 36–42.

[ca70031-bib-0003] Altenberg, A. R. 1977. “Acetabular Labrum Tears: A Cause of Hip Pain and Degenerative Arthritis.” Southern Medical Journal 70, no. 2: 174–175. 841394

[ca70031-bib-0004] Arnoczky, S. P. , and R. F. Warren . 1982. “Microvasculature of the Human Meniscus.” American Journal of Sports Medicine 10, no. 2: 90–95. 10.1177/036354658201000205 7081532

[ca70031-bib-0005] Barber‐Westin, S. D. , and F. R. Noyes . 2014. “Clinical Healing Rates of Meniscus Repairs of Tears in the Central‐Third (Red‐White) Zone.” Arthroscopy 30, no. 1: 134–146. 10.1016/j.arthro.2013.10.003 24384277

[ca70031-bib-0006] Battistelli, M. , E. Tassinari , G. Trisolino , et al. 2023. “Hip Labral Morphological Changes in Patients With Femoroacetabular Impingement Speed up the Onset of Early Osteoarthritis.” Calcified Tissue International 112, no. 6: 666–674. 10.1007/s00223-023-01076-1 PMC10199105 36949181

[ca70031-bib-0007] Beaufils, P. , and N. Pujol . 2017. “Management of Traumatic Meniscal Tear and Degenerative Meniscal Lesions. Save the Meniscus.” Orthopaedics & Traumatology, Surgery & Research 103, no. 8s: S237–S244. 10.1016/j.otsr.2017.08.003 28873348

[ca70031-bib-0008] Bednar, M. S. , S. P. Arnoczky , and A. J. Weiland . 1991. “The Microvasculature of the Triangular Fibrocartilage Complex: Its Clinical Significance.” Journal of Hand Surgery 16, no. 6: 1101–1105. 10.1016/s0363-5023(10)80074-7 1748756

[ca70031-bib-0009] Benjamin, M. , and E. J. Evans . 1990. “Fibrocartilage.” Journal of Anatomy 171: 1–15. PMC1257123 2081696

[ca70031-bib-0010] Benjamin, M. , H. Toumi , J. R. Ralphs , G. Bydder , T. M. Best , and S. Milz . 2006. “Where Tendons and Ligaments Meet Bone: Attachment Sites ('entheses') in Relation to Exercise and/or Mechanical Load.” Journal of Anatomy 208, no. 4: 471–490. 10.1111/j.1469-7580.2006.00540.x PMC2100202 16637873

[ca70031-bib-0011] Bharam, S. 2006. “Labral Tears, Extra‐Articular Injuries, and Hip Arthroscopy in the Athlete.” Clinics in Sports Medicine 25, no. 2: 279–292. 10.1016/j.csm.2006.01.003 16638491

[ca70031-bib-0012] Bsat, S. , H. Frei , and P. E. Beaule . 2016. “The Acetabular Labrum: A Review of Its Function.” Bone and Joint Journal 98‐B, no. 6: 730–735. 10.1302/0301-620X.98B6.37099 27235512

[ca70031-bib-0013] Byers, P. D. , C. A. Contepomi , and T. A. Farkas . 1970. “A Post Mortem Study of the Hip Joint. Including the Prevalence of the Features of the Right Side.” Annals of the Rheumatic Diseases 29, no. 1: 15–31. 10.1136/ard.29.1.15 PMC1031217 5416096

[ca70031-bib-0014] Carter, D. R. 1987. “Mechanical Loading History and Skeletal Biology.” Journal of Biomechanics 20, no. 11: 1095–1109. 10.1016/0021-9290(87)90027-3 3323201

[ca70031-bib-0015] Cooper, D. E. , S. P. Arnoczky , S. J. O'Brien , R. F. Warren , E. DiCarlo , and A. A. Allen . 1992. “Anatomy, Histology, and Vascularity of the Glenoid Labrum. An Anatomical Study.” Journal of Bone and Joint Surgery. American Volume 74, no. 1: 46–52. 1734013

[ca70031-bib-0016] Cotten, A. , N. Boutry , X. Demondion , et al. 1998. “Acetabular Labrum: MRI in Asymptomatic Volunteers.” Journal of Computer Assisted Tomography 22, no. 1: 1–7. 10.1097/00004728-199801000-00001 9448753

[ca70031-bib-0017] Di Giancamillo, A. , D. Deponti , S. Modina , I. Tessaro , C. Domeneghini , and G. M. Peretti . 2017. “Age‐Related Modulation of Angiogenesis‐Regulating Factors in the Swine Meniscus.” Journal of Cellular and Molecular Medicine 21, no. 11: 3066–3075. 10.1111/jcmm.13218 PMC5661103 28580627

[ca70031-bib-0018] Domzalski, M. E. , M. Synder , A. Karauda , and W. Papierz . 2017. “Histological Changes of the Acetabular Labrum in Coxarthrosis: Labral Degeneration and Repair.” Hip International 27, no. 1: 66–73. 10.5301/hipint.5000425 27834459

[ca70031-bib-0019] Fuller, E. S. , M. M. Smith , C. B. Little , and J. Melrose . 2012. “Zonal Differences in Meniscus Matrix Turnover and Cytokine Response.” Osteoarthritis and Cartilage 20, no. 1: 49–59. 10.1016/j.joca.2011.10.002 22062355

[ca70031-bib-0020] Harris, W. H. , R. B. Bourne , and I. Oh . 1979. “Intra‐Articular Acetabular Labrum: A Possible Etiological Factor in Certain Cases of Osteoarthritis of the Hip.” Journal of Bone and Joint Surgery. American Volume 61, no. 4: 510–514. 438237

[ca70031-bib-0021] Heo, Y. S. , and H. J. Song . 2011. “Characterizing Cutaneous Elastic Fibers by Eosin Fluorescence Detected by Fluorescence Microscopy.” Annals of Dermatology 23, no. 1: 44–52. 10.5021/ad.2011.23.1.44 PMC3119998 21738362

[ca70031-bib-0022] Hunt, D. , J. Clohisy , and H. Prather . 2007. “Acetabular Labral Tears of the Hip in Women.” Physical Medicine and Rehabilitation Clinics of North America 18, no. 3: 497–520 ix‐x. 10.1016/j.pmr.2007.05.007 17678764

[ca70031-bib-0023] Ito, K. , M. Leunig , and R. Ganz . 2004. “Histopathologic Features of the Acetabular Labrum in Femoroacetabular Impingement.” Clinical Orthopaedics and Related Research 429: 262–271. 10.1097/01.blo.0000144861.11193.17 15577497

[ca70031-bib-0024] Kawamura, Y. , T. Tetsunaga , K. Yamada , et al. 2021. “Mechanical Stretching Induces Calcification and Cartilage Matrix Metabolism, Causing Degeneration of the Acetabular Labrum.” Hip International 33, no. 3: 500–507. 10.1177/11207000211044675 34538120

[ca70031-bib-0025] Kelly, B. T. , G. S. Shapiro , C. W. Digiovanni , R. L. Buly , H. G. Potter , and J. A. Hannafin . 2005. “Vascularity of the Hip Labrum: A Cadaveric Investigation.” Arthroscopy: The Journal of Arthroscopic & Related Surgery 21, no. 1: 3–11. 10.1016/j.arthro.2004.09.016 15650660

[ca70031-bib-0026] Koch, S. , and B. Tillmann . 1995. “The Distal Tendon of the Biceps Brachii. Structure and Clinical Correlations.” Annals of Anatomy 177, no. 5: 467–474. 10.1016/S0940-9602(11)80155-X 7645742

[ca70031-bib-0027] Koo, T. K. , and M. Y. Li . 2016. “A Guideline of Selecting and Reporting Intraclass Correlation Coefficients for Reliability Research.” Journal of Chiropractic Medicine 15, no. 2: 155–163. 10.1016/j.jcm.2016.02.012 PMC4913118 27330520

[ca70031-bib-0028] Lecouvet, F. E. , B. C. Vande Berg , J. Malghem , et al. 1996. “MR Imaging of the Acetabular Labrum: Variations in 200 Asymptomatic Hips.” AJR. American Journal of Roentgenology 167, no. 4: 1025–1028. 10.2214/ajr.167.4.8819406 8819406

[ca70031-bib-0029] Lewis, C. L. , and S. A. Sahrmann . 2006. “Acetabular Labral Tears.” Physical Therapy 86, no. 1: 110–121. 10.1093/ptj/86.1.110 16386066

[ca70031-bib-0030] McCarthy, J. , P. Noble , F. V. Aluisio , M. Schuck , J. Wright , and J. A. Lee . 2003. “Anatomy, Pathologic Features, and Treatment of Acetabular Labral Tears.” Clinical Orthopaedics and Related Research 406: 38–47. 10.1097/01.blo.0000043042.84315.17 12578998

[ca70031-bib-0031] McCarthy, J. C. , and B. Busconi . 1995. “The Role of Hip Arthroscopy in the Diagnosis and Treatment of Hip Disease.” Orthopedics 18, no. 8: 753–756. 10.3928/0147-7447-19950801-12 7479416

[ca70031-bib-0032] McCarthy, J. C. , P. C. Noble , M. R. Schuck , J. Wright , and J. Lee . 2001. “The Otto E. Aufranc Award: The Role of Labral Lesions to Development of Early Degenerative Hip Disease.” Clinical Orthopaedics and Related Research 393: 25–37. 10.1097/00003086-200112000-00004 11764355

[ca70031-bib-0033] Mikic, B. , T. L. Johnson , A. B. Chhabra , B. J. Schalet , M. Wong , and E. B. Hunziker . 2000. “Differential Effects of Embryonic Immobilization on the Development of Fibrocartilaginous Skeletal Elements.” Journal of Rehabilitation Research and Development 37, no. 2: 127–133. 10850818

[ca70031-bib-0034] Mulchrone, K. F. , and K. R. Choudhury . 2004. “Fitting an Ellipse to an Arbitrary Shape: Implications for Strain Analysis.” Journal of Structural Geology 26, no. 1: 143–153.

[ca70031-bib-0035] Nakano, T. , C. M. Dodd , and P. G. Scott . 1997. “Glycosaminoglycans and Proteoglycans From Different Zones of the Porcine Knee Meniscus.” Journal of Orthopaedic Research 15, no. 2: 213–220. 10.1002/jor.1100150209 9167623

[ca70031-bib-0036] Pauli, C. , S. P. Grogan , S. Patil , et al. 2011. “Macroscopic and Histopathologic Analysis of Human Knee Menisci in Aging and Osteoarthritis.” Osteoarthritis and Cartilage 19, no. 9: 1132–1141. 10.1016/j.joca.2011.05.008 PMC3217905 21683797

[ca70031-bib-0037] Pauli, C. , R. Whiteside , F. L. Heras , et al. 2012. “Comparison of Cartilage Histopathology Assessment Systems on Human Knee Joints at All Stages of Osteoarthritis Development.” Osteoarthritis and Cartilage 20, no. 6: 476–485. 10.1016/j.joca.2011.12.018 PMC3348372 22353747

[ca70031-bib-0038] Petersen, W. , F. Petersen , and B. Tillmann . 2003. “Structure and Vascularization of the Acetabular Labrum With Regard to the Pathogenesis and Healing of Labral Lesions.” Archives of Orthopaedic and Trauma Surgery 123, no. 6: 283–288. 10.1007/s00402-003-0527-7 12802599

[ca70031-bib-0039] Petersen, W. , T. Pufe , B. Kurz , R. Mentlein , and B. Tillmann . 2002. “Angiogenesis in Fetal Tendon Development: Spatial and Temporal Expression of the Angiogenic Peptide Vascular Endothelial Cell Growth Factor.” Referate Und Beiträge Zur Anatomie Und Entwickelungsgeschichte 205, no. 4: 263–270. 10.1007/s00429-002-0241-1 12136256

[ca70031-bib-0040] Polito, U. , G. M. Peretti , M. Di Giancamillo , et al. 2020. “Meniscus Matrix Remodeling in Response to Compressive Forces in Dogs.” Cells 9, no. 2: 265. 10.3390/cells9020265 PMC7072134 31973209

[ca70031-bib-0041] Rohde, M. S. , S. Trivedi , S. Randhawa , et al. 2023. “Pediatric Meniscus Morphology Varies With Age: A Cadaveric Study.” Knee Surgery, Sports Traumatology, Arthroscopy 31, no. 10: 4179–4186. 10.1007/s00167-023-07447-3 37178242

[ca70031-bib-0042] Safran, M. R. 2010. “The Acetabular Labrum: Anatomic and Functional Characteristics and Rationale for Surgical Intervention.” Journal of the American Academy of Orthopaedic Surgeons 18, no. 6: 338–345. 10.5435/00124635-201006000-00006 20511439

[ca70031-bib-0047] Schon, J. , J. Chahla , S. Paudel , et al. 2020. “Expression Profile of Matrix Metalloproteinases in the Labrum of Femoroacetabular Impingement.” Bone Joint Research 9, no. 4: 173–181. 10.1302/2046-3758.94.BJR-2019-0083.R1 PMC7229337 32431808

[ca70031-bib-0043] Seldes, R. M. , V. Tan , J. Hunt , M. Katz , R. Winiarsky , and R. H. Fitzgerald Jr. 2001. “Anatomy, Histologic Features, and Vascularity of the Adult Acetabular Labrum.” Clinical Orthopaedics and Related Research 382: 232–240. 10.1097/00003086-200101000-00031 11153993

[ca70031-bib-0044] Solis‐Cordova, J. , J. H. Edwards , H. L. Fermor , P. Riches , C. L. Brockett , and A. Herbert . 2023. “Characterisation of Native and Decellularised Porcine Tendon Under Tension and Compression: A Closer Look at Glycosaminoglycan Contribution to Tendon Mechanics.” Journal of the Mechanical Behavior of Biomedical Materials 139: 105671. 10.1016/j.jmbbm.2023.105671 36682172

[ca70031-bib-0045] Ueo, T. , and M. Hamabuchi . 1984. “Hip Pain Caused by Cystic Deformation of the Labrum Acetabulare.” Arthritis and Rheumatism 27, no. 8: 947–950. 10.1002/art.1780270817 6466398

[ca70031-bib-0046] Yan, W. , W. Dai , J. Cheng , et al. 2021. “Advances in the Mechanisms Affecting Meniscal Avascular Zone Repair and Therapies.” Frontiers in Cell and Development Biology 9: 758217. 10.3389/fcell.2021.758217 PMC8581462 34778268

